# Medical student's attitude towards learning medical ethics in India

**DOI:** 10.6026/973206300220566

**Published:** 2026-01-31

**Authors:** Vino Tito V Kurien, Poonam R Naik, Mahitha M.C, Bhargav Sunkara, Amrita Das, Siva A.S Pillai

**Affiliations:** 1 Department of Dentistry, Dr. Somervell Memorial CSI Medical College & Hospital , Karakonam P.O, Thiruvananthapuram, Kerala, India; 2 Department of Community Medicine, Yenepoya Medical College, Mangalore, India; 3 Department of Community Medicine, Dr. Somervell Memorial CSI Medical College & Hospital, Karakonam P.O, Thiruvananthapuram, Kerala, India; 4 Department of Orthopaedics, Yashodha Hospitals, Hyderabad, India; 5 Department of Periodontology , Maharana Pratap College of Dental Sciences and Research Centre, Gwalior, Madhya Pradesh, India; 6 Department of Dentistry, Asmat Dental Medical Center, Abu Dhabi, United Arab Emirates

**Keywords:** Medical ethics, medical students, survey, attitude

## Abstract

The role of medical ethics in healthcare is crucial. However, its absence from many institutional curricula has resulted in a considerable vacuum in professional education. This cross-sectional study of 90 first-year MBBS show discovered that all participants recognized the importance of ethics education, with 90% believing it is essential for providing good patient care. Furthermore, the majority of students argued for the horizontal and vertical integration of ethics throughout their medical education. Even individuals with little prior experience with formal ethics training had a good attitude. Thus, innovative, integrated ethics program should be established beginning in the first year of medical school to better prepare students for the ethical problems of daily practice.

## Background:

The term "Ethics" has been described as "the moral principles that govern a person's behaviour or how an activity is carried out," while "medical ethics" has been defined as "the branch of knowledge concerned with moral principles." The application of ethics in medical practice dates back to ancient civilizations and even today, all medical graduates must swear symbolic adherence to the Hippocratic Oath. Codes of behaviour and laws governing the medical profession are periodically established [1]. Worldwide, medical practice has grown increasingly commercialized and ethics has taken a back seat. It has been demonstrated that teaching ethics has a major impact on the professionalism and moral traits of medical personnel [2]. The goal of teaching ethics is to help students recognize and deal with difficult situations in a more rational and ethical manner. Ethics training is designed to assist medical students comprehend and assess moral ideas, as well as form their own beliefs. The public is becoming more aware of medical practitioners' ethical behaviour and complaints against physicians appear to be on the rise. In today's highly complex and costly medical therapy, healthcare personnel' knowledge and application of medical ethics is crucial. Medical ethics, which has its roots in early Christian teachings and the Hippocratic Oath, is essentially the application of moral norms and principles to the practice of medicine. Autonomy, beneficence, justice and no maleficence are the four basic tenets of ethical medical practice [3, 4]. The Medical Council of India (MCI), the regulating organization for medical education, has mandated medical ethics courses in the undergraduate curriculum. MCI published its code of ethics in 2002, which served as a regulating framework for doctors' professional conduct, etiquette and ethics [5]. Nonetheless, student responses, perspectives and comprehension are critical to ensuring the delivery of a curriculum that is relevant and interesting to them [6]. Furthermore, the student's perspective is seen as a significant component in the evaluation of medical education [7]; so, students should be requested to submit feedback in order to change the curriculum based on their requirements and interests [8]. Teaching medical ethics will surely raise students' awareness and knowledge of the importance of the human side of medicine. It will also offer students with an effective technique for recognizing and analyzing ethical dilemmas that may arise in clinical practice, both in terms of curriculum content and implementation, as well as proper evaluation [9]. Several studies have demonstrated the need of including ethical and legal problems in medical education. Medical students are taught a variety of disciplines to help them deal with medical concerns; they also need ethics to help them overcome moral quandaries that they will most likely encounter in their future profession. Medical ethics are vital to all clinical encounters and public health campaigns, thus students must have a solid understanding of medical ethics in order to become virtuous doctors [10]. Therefore, it is of interest to evaluate the attitude for learning medical ethics among medical students in India.

## Methodology:

A cross-sectional study was conducted in teaching institute during the academic year 2019-2020, from October 2019-November 2019. A self- administered questionnaire was given to the first-year undergraduates of MBBS students of teaching institute. 90 questionnaires were distributed to first year MBBS students of a private medical college in Mangalore and collected back. Data was entered into the SPSS version software and analyzed using its data analysis programmes. Written informed consent was obtained from 90 students and they were included in this study. Confidentiality was maintained and participants were guaranteed anonymity. In addition, data were kept secured. This study was approved by Research Ethics Committee (YEC2/168). After obtaining permission from Institutional ethics committee, the principal investigator distributed the questionnaire to the first year MBBS students. The questionnaire included attitudes towards learning medical ethics in the new curriculum consisted of 15 items. The answers of the students were categorized in to groups: Agree, Disagree, Neither Agree or Disagree, Yes, No. The purpose of the study and nature of information which has to be furnished from the students was explained to them. Willingness to participate in the study was obtained from participants using written consent form. It was ensured that participants understood the meaning of questions as well.

## Results:

90 Questionnaires were collected from the participants. All the participants agreed that medical ethics teaching for medical students is an important aspect of medical education. 96.7% of the participants agreed that teaching medical ethics would influence the attitude and behaviour of doctors and improve patient-doctor relationship (Table 1). 80% of the participants disagreed that medical ethics should be taught only through lectures (Table 1). An interest in learning more about medical ethics was seen in around 86.7% of the participants (Table 2).

## Discussion:

The term 'Medical Ethics' is attributed to the English physician Thomas Percival, who in the year 1803 first published the expectations and requirements of a member of the medical profession. It is based on a set of values that healthcare practitioners should rely on in case of conflict or confusion and includes four main principles [3]. This study aimed to assess medical students' attitudes regarding incorporation of medical ethics in medical curriculum in a teaching institute. In our study, all the participants responded which reflects the positive attitudes of students towards medical ethics. In our study all the participants' agreed that teaching medical ethics is an important aspect in the new curriculum. 60% of the participants said that they received some type of teaching on ethics before joining MBBS course. The source of knowledge may be from seminars/lectures or personal training in schools. Roberts *et al.* study carried out in 2004 at the University of New Mexico School of Medicine, the respondents strongly proved the appropriateness of ethics education which improved clinical care and interpersonal skills [11]. In our study nearly 67.8% of the students agreed that medical ethics teaching should be incorporated horizontally and vertically within the medical curriculum. A higher incidence was reported in a study conducted in 2006 at Queen Elizabeth Hospital in Barbados, which revealed that medical students were extremely hopeful about the relevance of ethical awareness [12]. In 2007, Johnston and Haughton of King's College, London School observed that the majority of medical students found the subject to be very relevant, with half of them engaged in medical ethics instruction. As a result, teaching medical ethics to students will benefit their research endeavors [6]. In our study, 73.7% of students agreed that ethical knowledge is vital for them as researchers. During clinical years of training, a more efficient manner of giving ethics instruction could be for an ethics expert and a clinical expert to co-lead ethics clerkship sessions, as has been successfully implemented at the Icahn School of Medicine at Mount Sinai in the United States [13]. In our research, 80% of students disagreed that medical ethics should not be taught solely through lectures. According to Ramesh Acharya and colleagues' research, students learned (35.7%) from lectures/seminars, (24.5%) on the job and (21.4%) through training. Shiraz *et al.* Obtained comparable results in a study of surgical residents and interns in Pakistan [14]. Concerning the importance of understanding medical ethics to help people detect ethical difficulties in their future profession, the clear majority (96.7%) agreed with the statement. In a Barbados poll, all physicians and 90% of nursing staff agreed that knowledge of ethics is crucial in their profession [15]. A study conducted at the University of New Mexico in 2009 found that medical students agreed that medical ethics education helps practitioners detect ethical difficulties, clarify "value- laden choices", improve patient care and make clinical decisions [16]. In our study, 90% of participants believed that medical ethics education in a formal setting is critical to providing appropriate patient care. Another question questioned about interest in learning medical ethics; 86.7% of participants expressed a general interest in learning medical ethics. Similarly, a 2010 study conducted by King Saud bin AbdulAziz University for Health Sciences Indicated that most students agreed strongly on the significance of learning biomedical ethics, with half finding the topic intriguing [17]. An undergraduate school at West Bengal Medical agreed that learning medical ethics was critical for future jobs [2]. Chin *et al.* (2011) discovered that a third to a quarter of students thought ethics education was an important part of medical education [18]. According to a study conducted by Lafta *et al.* students chose clinically integrated and case studies with discussion groups, followed by role play and regular lecturing was last [9]. According to our research, 96.7% of students believe that ethical case conferences are necessary for learning more about ethical issues. Another 2004 study by Roberts *et al.* found that respondents rated clinical approaches (faculty role modelling, clinical rounds, interaction with patients and case conferences) as the most effective, while formal didactic approaches (grand round presentation and lectures) were only moderately effective [11]. Students at New Mexico University strongly agreed that ethics training should involve group discussions, advice and guidance on the ethical and scientific designs of specific procedures, as well as engagement with institutional review [16]. The modern workplace in general medical practice is stressful and demanding and the job of a consulting doctor is difficult. In our survey, 90% of undergraduates felt that medical ethics education would increase their understanding of the complexities of medical practice. So, in general, physicians must be cognizant of the ethical and legal implications of modern medicine, which permeate all areas of their work. This will prepare them to face practical ethical concerns during their work. In our survey, 96.7% of students believed that teaching medical ethics will impact doctors' attitudes and behaviour while also improving the patient-doctor interaction. So, mastering medical ethics may serve as a means of sensitizing clinicians, so bridging the gap between patient and doctor. Such an initiative will result in a more amicable doctor-patient relationship. While regulatory codes offer a framework, the shift to a structured, longitudinal curriculum like AETCOM (Attitude, Ethics and Communication) is essential to go beyond theoretical awareness toward practical ability, as evidenced by the persistence of ethical knowledge gaps among graduates [19]. Despite the fact that the majority of medical students support these courses [20], their preference for interactive clinical mentorship and narrative reflection over didactic lectures highlights the necessity for stakeholders to give practical, small-group involvement top priority [21, 22].

Additionally, the long-term development of academic ethics seen here indicates that gender-sensitive curricula are required to guarantee integrated professional identity construction due to sex-based disparities in peer perception [23]. In the end, even though students appreciate the new curriculum, its successful implementation necessitates resolving systemic issues including passive learning, logistical limitations and mental exhaustion in order to strike a balance between the demanding competency standards and sufficient time for independent study [24, 25]. In our questionnaire, most medical students had a good view toward medical ethics teaching. We could assess undergraduate medical students' views toward medical ethics to gather fundamental information for the development of a bioethics course in the medical curriculum. It would be useful to include a bioethics curriculum in the early stage of graduation and postgraduate studies.

## Conclusion:

Medical ethics should be included into the curriculum beginning in the first year, employing problem-based learning and small-group case studies instead of standard lectures. Schools should use a variety of teaching methodologies, including ethical portfolios and seminars, while boosting the subject's weight in summative exams. However, larger-scale study required to generalize these findings and further assess student grasp of professional responsibility.

## Figures and Tables

**Table 1:** Students attitude in learning medical ethics in new curriculum- answers interpreted for agree, disagree, neither agree nor disagree [9].

**Questions**	**AGREE (%)**	**DISAGREE (%)**	**NEITHER AGREE NOR DISAGREE (%)**
Q1)Medical ethics teaching for medical students is important aspect of medical education	90(100)	0(0)	0(0)
Q2) Medical ethics education in a formal course is crucial to good patient care.	81(90)	3(3.3)	6(6.7)
Q3) Medical ethics teaching should be integrated horizontally and vertically within the medical curriculum	61(67.8)	5(5.6)	24(26.7)
Q4) Medical teachers need to have formal education in Medical ethics	77(85.6)	1(1.1)	12(13.3)
Q5) Ethics knowledge is important to me as a medical teacher	81(90)	0(0)	9(10)
Q6) Ethics knowledge is important to me as a researcher	66(73.3)	1(1.1)	23(25.6)
Q7) Medical ethics education will make me more aware of the complexity of the practice of medicine	81(90)	3(3.3)	6(6.7)
Q8) Medical ethics teaching would influence the attitude and behaviour of doctors and improve patient-doctor relationship	87(96.7)	2(2.2)	1(1.1)
Q9) Ethical conduct is important only to avoid legal action	27(30)	52(57.8)	11(12.2)
Q10) Medical ethics should be taught only through lectures	9(10)	72(80)	9(10)
Q11) Learning Medical ethics would help me to identify ethical issues in my future work	87(96.7)	0(0)	3(3.3)
Q12) Adequate opportunity to confront practical ethical issues during the course is also important	88(97.8)	0(0)	2(2.2)
Q13) Ethical case conferences are important to know more about ethical issues	87(96.7)	0(0)	3(3.3)

**Table 2:** Students attitude in learning medical ethics in new curriculum-answers interpreted for Yes, No [9]

**Questions**	**NO (%) (%)**	**YES (%) (%)**
Q1) I have interest in learning more about medical ethics	12(13.3)	78(86.7)
Q2) Did you receive any type of teaching on ethics before joining MBBS course?	36(40)	54(60)
